# Shooting Yourself First in the Foot, then in the Head: Normative Democracy Is Suffocating, and then the Coronavirus Came to Light

**DOI:** 10.1007/s42438-020-00142-3

**Published:** 2020-06-10

**Authors:** Paul R. Carr

**Affiliations:** Département des sciences de l’éducation & Chair-holder, UNESCO Chair in Democracy, Global Citizenship and Transformative Education (DCMÉT), Université du Québec en Outaouais (UQO), Gatineau, Québec Canada

**Keywords:** Normative democracy, Coronavirus, Covid-19, Hegemony, Social media, Social inequalities, Citizen engagement, Neoliberalism, Québec

## Abstract

This text starts with the premise that ‘normative democracy’ has rendered our societies vulnerable and burdened with unaddressed social inequalities. I highlight three central arguments: (1) Social media, and, consequently, citizen engagement are becoming a significant filter that can potentially re-imagine the political, economic, and social worlds, which increasingly bleed over to how we might develop and engage with ‘democracy’; to this end, I introduce a brief case study on the nefarious interpretation of the killing of Jamal Khashoggi in 2018 to underscore the tension points in normative democracy; (2) Capitalism, or neoliberalism, needs to be more fully exposed, interrogated, and confronted if ‘normative, representative, hegemonic, electoral democracy’ is to be re-considered, re-imagined, and re-invented; the perpetuation of social inequalities lays bare the frailty of normative democratic institutions; (3) Covid-19 has exposed the fault lines and fissures of normative democracy, illustrating here the ‘common sense’ ways that power imbalances are sustained, which leaves little room for social solidarity; I present herein the case of the economic/labor dynamic in Quebec during the coronavirus. Ultimately, I believe the quest to re-imagine a more meaningful, critically engaged democracy, especially during a context that is imbued with a political, economic, and public health crisis, cannot be delayed much longer.

## Introduction

We are taught to believe that we live in a democracy, that we have freedom, free speech and all sorts of rights and liberties, and that we are in control of our destiny. It is helpful to deconstruct every contour, nuance, concept, and meaning of democracy. To start, the ‘democracy’ I am referring to above is the larger-than-life, win-at-all-costs, ‘normative, representative, hegemonic, electoral democracy’, the one with the, generally speaking, two main political parties, highly controlled elections, representatives who, in some way, (supposedly) represent constituents, the common legislative, administrative, and judicial institutions that are supposed to be neutral and devoid of political influence, and the other trappings that give the sense that one would have to be literally insane to not endorse such a regime (Carr and Thésée 2019). Some might argue that we have no alternative, that we have no way out, and that this system that we have been led to believe is so fundamentally essential to our being cannot be rivaled by anything else. This *normative* democracy is considered to be the standard, the norm, the indisputable, unquestionable system that allows us to live, grow, prosper, and function. With this normative understanding of democracy, it can be difficult to introduce opposing, counter and/or critical proposals, philosophies, and changes that challenge the formal political structures and regimes. To be clear, this vision and version of normative democracy does not infer that historically marginalized peoples (starting with the First Nations and African-Americans within the North American context) necessarily support formal power structures; rather, it is important to underscore that they, in general, continue to be further victimized by a system that upholds significant power differentials.

So I stand on the outside of this behemoth monolith that has so shaped and dominated our realities and experiences. I am a part of a system that I feel uncomfortable with (like many other people), yet the space to re-imagine and re-invigorate something different is tightly and hermeneutically sealed over our minds, requiring serious acrobatic contortions to overcome. Again, vast social inequalities make supporting normative democracy an uneasy and debilitating option for broad swaths of the population but the normative structure and system in place does not lead to a plethora of openings and options to build something more engaging. To prove the point—or at least to expose it— we can ask how easy it is to speak (within broad, mainstream sectors of society) of developing a different, alternative democracy, especially during election season, which seems to be increasingly all of the time (Masket 2019)? Normative democracy, not the philosophical, political-science versions, is imbued in what could be considered ‘liberal democracy’, and this formulation/conceptualization has been led, principally, by the Western world, and, especially the USA.

After some 15 years of researching the meaning, manifestation and operationalization of democracy, I do not think that it is unfair to submit that for many people democracy should naturally equal the ‘normative, representative, hegemonic, electoral democracy’ that is suffocating humanity. This is neither to say that most people are satisfied, uplifted, and enthralled with the status quo, nor to say that they are ignorant, disengaged and incapable of effectuating change. Rather, it underscores how difficult and even unimaginable it is to consider another form of democracy. The linkage with education and education for democracy is key to this debate (Apple 2018; Thésée, Carr, Duclos, and Potwora 2018), and contextualizes how we think (and act) about the subject.

To further clarify this context of how normative democracy manifests itself, I highlight the following examples from my research projects with colleagues over the years, which involved studies in some 15 countries with roughly 5000 teacher education and educator participants (Carr and Thésée 2019):

- we found that the vast majority did not have a robust, significant democratic experience in their own education;
- that this has affected how they consider democracy and education;
- that social justice is, for many, a difficult and problematic area to cultivate in and through education owing to a weakly asserted, structured, and supported institutional culture based on normative democracy;
- that most considered that the space for inclusive and critical engagement in and through education is constrained, limited, and fraught with obstacles;
- that racialized participants had significantly higher levels of experience, conscientization, and engagement with, for example, racism, antiracism, and efforts to address racial inequities, which further underscores how normative democracy closes down fundamental debate, dialog, teaching and learning as well as transformative education while presenting the posture and framework of democracy.

When democracy and education are considered to be naturally disconnected while not leaving room for a more critically engaged democracy, it is not difficult to imagine the suffocating nature of normative democracy.

Normative elections, the ones that have been so effectively presented by the USA as the backbone to any meaningful democracy, have been jettisoned into a cesspit of turmoil and intractable debate that often neglects problematizing some of the most intractable and germane issues (Achen and Bartels 2017; Howe 2010; Torcal and Ramon Montero 2013). Not everyone involved in elections is corrupt or corrupted, or is afflicted with unsightly motivations, and people who go to the polls are not simply sheep being led to the proverbial slaughterhouse. There is a great deal of complexity as to why we vote and why we hope that there will be some hope in participating in mainstream democracy, but the faith in electoral democracy is waning almost everywhere (Torcal and Ramon Montero 2013; Carr and Thésée 2019). Yet, these normative elections, which are often ordered to measure with the threat of massive (real and rhetorical) carpet-bombing and worse, if not realized, are replete with all kinds of paradoxical anti-democratic maneuvers, starting with who can be elected, how much money plays into the process, how media can control and shape the message, manipulation, and diversion is a fundamental component, how seeking to win is more a priority than seeking to build a meaningful democracy, and how capitalism is the enormous, indelicate, meandering proverbial 800-pound gorilla in the room (Amico 2020; Carr and Thésée 2019). Added to this is the role, the purpose, and place of education in supporting, cultivating, and building a critically engaged democracy as well as critically engaged citizen participation. It is extremely difficult to have one without the other (democracy without education, for example, or, rather, meaningful, critically engaged democracy without meaningful, critically engaged education). (See Carr 2011, and Carr and Thésée 2019, as well as the UNESCO Chair DCMÉT website at uqo.ca/dcmet/ for an archive of publications.).

And then, starting in late 2019, the world started to feel the indelible, intractable, and (in)visible perturbations of the coronavirus, which emanated in China, and has quickly disseminated throughout all regions, making it a global pandemic. The number of people affected, contracting the virus, and ultimately succumbing to it, is increasing daily at this time but there is much analysis and data-crunching indicating that, in many areas, after several weeks of self-distancing, hygienic measures, increasing testing, closing down all but ‘essential services’, and enhancing medical and health care measures, the ‘curve’ may be flattening. However, few people believe that the virus will disappear, nor that the cost, in terms of human life, will be entirely negligible. So what is the connection to democracy, capitalism (or perhaps more correctly neoliberalism), and Covid-19? The vulnerabilities, inequalities, and fault lines that existed prior to the Coronavirus have been exacerbated, and the virus has disproportionately impacted racialized, marginalized, and lower income communities. The contraction and death rates are higher, and the economic, labor, living, and social conditions have worsened, notably for already vulnerable communities. This pandemic, sadly, provides a tremendous and significant impetus to re-consider and re-calibrate our thinking around democracy (Diamond 2020; Giroux 2020; Roy 2020).

This text starts with the premise that ‘normative democracy’ has put us in a pickle, and that, although there are ways out if it, this will require breaking out of the glass box that has a great many of us believing that there is no alternative. I highlight three points related to democracy in this text, formulating the following central arguments:

1. Social media and, consequently, citizen engagement are becoming a significant filter that can potentially re-imagine the political, economic, and social worlds (outside of and beyond normative democracy), which increasingly bleed over to how we might develop and engage with ‘democracy’ (Garrett 2019); to this end, the advent of ‘fake news’ is a worthy subject to explore here because a functioning democracy, to a certain degree, is dependent on media/political literacy, critical engagement/participation, and the capacity to communicate, analyze, and disseminate nuanced perspectives, ideas, and information; I introduce a brief case study on the nefarious interpretation of the killing of Jamal Khashoggi in 2018 (BBC News 2019) to underscore the tension points in normative democracy;
2. Capitalism, or neoliberalism, needs to be more fully exposed, interrogated, and confronted if ‘normative, representative, hegemonic, electoral democracy’ is to be re-considered, re-imagined, and re-invented (Lydon 2017); the perpetuation of social inequalities lays bare the frailty of normative democratic institutions;
3. Covid-19 has exposed the fault lines and fissures of normative democracy, illustrating here the ‘common sense’ ways that power imbalances are sustained, which leaves little room for social solidarity (Human Rights Watch 2020); I present here a small case study of the economic and labor dynamic in Quebec during the coronavirus.

Ultimately, I believe the quest to re-imagine a more meaningful, critically engaged democracy, especially during a context that is imbued with a political, economic, and public health crisis, cannot be delayed much longer.

### Social Media and Citizen Engagement

Capitalism, in addition to acknowledged and unacknowledged hegemony, is central to this model or framework, and a natural order and superiority flows effortlessly through thinking and believing that *this* is the only way to be, exist and function. Democracy 2.0, which considers more fluidly agency, power crystallizations, social justice, and individual as well as collectivist media and citizen engagement, is much messier than Democracy 1.0, which connects more directly with normative, representative, hegemonic, and electoral machinations (Carr, Hoechsmann, and Thésée 2018). Social media is an exemplary feature of this new environment and can help us draw out the fundamental question if greater media, communication, and online involvement can lead to more robust, critical democratic forms of citizen participation. Elsewhere, with colleagues (Carr, Daros, Cuervo, and Thésée 2020), I describe some of the overlapping components, processes, and concerns that help frame the context for social media, fake news, and citizen participation.

It would appear that everyone today is somehow connected to social media, even if one does not have an account for one or many of the social networks that pervade, link and smother the socio-cultural landscape (Keating and Melis 2017). There are networks for an untold array of information sharing and gathering. Nouns have become verbs as in ‘youtubbing,’ ‘blogging,’ ‘vlogging,’ ‘googling,’ ‘facebooking,’ etc.. The reach is significant, and the digital imprints (and footprints) are equally commensurate (Sun, Wang, Shen and Zhang 2015). One can do a search for a pair of shoes on Amazon.com, and, magically, there will be ads for shoes on the personal Facebook feed immediately afterward. Algorithms are increasingly programming what we see, and aligning at least some of our attention on ‘stuff,’ for lack of a better word, where we might not otherwise be interested. This surveillance, usurpation and data-gathering was significantly exposed in 2018, with Facebook being highlighted for a particularly negative watershed year (Sutton 2018; Wong and Morris 2018). Among the litany of events, problems and phenomena that have plagued Facebook, which are clearly not limited to this one, albeit prominent social network, were the following claims, findings and evidence, amongst other issues: algorithms connected to the ‘negative effects to referral traffic,’ unregulated ads that underscored the Mueller investigation that has, as it focus, in large part, the Russian involvement in the 2016 US presidential election, the Cambridge Analytica scandal that ‘obtained the data of tens of millions of Facebook users without their knowledge or consent to help build a powerful political influencing tool,’ privacy and security issues, ‘special data-sharing arrangements with tech manufactures like Amazon, Apple and Samsung,’ hacking of accounts, and regulation problems (Sutton 2018). (Carr et al. 2020: 4)

Fake news has leaped into mainstream consciousness over the past few years as if *it* is the problem hampering democracy. Emphasizing that fake news is rarely neatly packaged within a singular category, the report cited above cautions that deception needs to be interrogated at various times while viewing media messages. With the avalanche of fake accounts, fake (bot) users, and fake (or tampered with) algorithms, the terrain is fertile for fake news. This is especially the case if users, consumers, and citizens are conditioned to not question or verify what comes their way, are reluctant to disbelief ‘official’ sources, are ignorant, are disinterested, or are enveloped in turbulent news cycles with complex, nuanced, voluminous information, for which they are unable to decipher the diverse and divergent realities emanating from a particular situation, event, or reality (Carr, Daros, Cuervo, and Thésée 2020). Citizen participation requires critical engagement, and constructing media/political literacy, however defined, needs to be considered in order to better underpin meaningful forms of democracy (Carr, Cuervo, and Daros 2019). The hailstorm of misinformation, misdirection, and disinformation during the early phases of the coronavirus mirrors the general online landscape, serving as both a tremendous opportunity and a mud-slide concurrently, and highlighting the potential for meaningful solidarity as well as, conversely, marginalization and xenophobia (Ali and Kurasawa 2020).

I am also drawn to the nuanced layers that MacKenzie and Bhatt (2020) add to this debate, suggesting that ‘[b]ullshit is different from lying and it need not undermine trust, particularly when it is blatant’. (The literature around the notion and proliferation of ‘bullshit’ is linked to, and builds on, the work of Harry G. Frankfurt, notably the 2005 book aptly entitled *On Bullshit*.) This is extremely relevant in contemporary times, given populist movements, xenophobic manifestations, and the denunciation of human rights, and the quest to diminish ‘news’ as being ‘fake’ as a basic principle emanating from some powerful leaders in the Western world as well as elsewhere. At the same time, I acknowledge that the traditional media is anchored in biases and hegemonic trappings but am troubled that the ‘fake news’ caravan seeks to whitewash anything that may bring contrary dimensions to the debate, especially in relation to revealing, exposing, and countering mainstream narratives related to war, conflict, racism, inequalities, and the like.

Democracy 1.0 had a relatively controlled audience, whereas Democracy 2.0 has let the floodgates open, and this means that there are now opportunities for critique, solidarity, and mobilization that may not have been as readily available previously, including diverse social movements that have taken off through social media (Carr, Daros, Cuervo, and Thésée 2020). These social movements can be a force of change in society at the local, national, and global levels where governments and international institutions are unwilling, unable, or unmotivated to respond to the needs of the population. For instance, Black Lives Matter (Mundt, Ross, and Burnett 2018), #metoo (Botti et al. 2019), Occupy, and Idle No More have all had a significant social media influence, and environmental, peace, and other movements have also been influential at diverse levels in mobilizing solidarity that surpasses cultural, linguistic, geographic, and political boundaries (Carr et al. 2020; Carty 2019).

#### Considering Killing as a Peripheral, Irrelevant Event, and the Death of a Saudi Journalist

Within the quickly evolving media/social media landscape, I can think of what appear to be several major (media and/or social) events in recent times—noting full-well that by the time this article is published, they may not even be recognizable—including the Khashoggi killing, the Covington School student debacle, the Parkland shooting, the Kavanaugh nomination, the Thai Cave recue, the (British) Royal wedding, the manifestations in Haiti, the never-ending quest to build a ‘wall/barrier/fence’ between the USA and Mexico, and the political/humanitarian crisis in Venezuela, among many others. I apologize for the USA-centric focus here. As a Canadian, I am fully cognizant of the depth and reach of USA tentacles, thinking, control, power, and influence in and on my own work, as well as on many others, even though I collaborate widely with colleagues in diverse jurisdictions and contexts, notably in Latin America. The USA and its interests bleed over to every region of the world, and, although United Statesians (the concept of ‘American’ is hotly contested and does not cover all of the peoples of the ‘Americas’) may not be talking collectively (in a central way) about the world or may not be collectively immersed in ingratiating the USA into the infinite number of political and economic issues, concerns, and cultural representations of the other countries and peoples, the world is watching, listening, and being consumed by the behemoth of USA empire.

The Covid-19 pandemic also squarely places the USA within the core of the action, with daily pronouncements about blaming China, cutting off funding to the World Health Organization, downplaying the spread of the virus, boasting about how the virus has been beaten back, and spreading the political and economic reach of this country far and wide, in military, diplomatic, commercial, and (potentially) humanitarian ways. It seems as though the reality of this being a (global) pandemic, a far-reaching health crisis, is only partially the story, and the present manifestations in the USA of people demanding that ‘isolation’ be stopped, while so many are being infected and even dying, is almost incomprehensible, and social media concurrently exposes, denounces, disseminates, and provides an echo chamber for what is taking place.

So I question what becomes news, indeed viral, and how does it become more than click-bait, algorithmic entertainment, the bouncing around in limited, like-minded networks, tepid sharing, and a platform for trolls? Is it about numbers, the quantity of clicks, views, shares and reads, or something more substantive? At the same time, what are the true dimensions of the issue(s)? Who frames it, how, and why, and to what end? What is omitted, downplayed, obfuscated, how and why? In the list of issues in the previous paragraph, we can think of many pitfalls, foibles, and problematic concerns as to what ‘issues’ look like in Democracy 2.0. All issues are not simply a USA problem, but connections to elites, hegemony, power differentials, and media framing are, I believe, worth establishing and interrogating. What is clear is that power differentials are at play in how fake news is constructed, disseminated, understood, and engaged with. The more volatile social media can push up against normative media in further determining how fake news can be projected, masked, embellished, and consumed.

Concerning the Jamal Khashoggi killing in October 2018 in Istanbul (BBC News 2019), we can follow the usual process of focusing on hegemonic interests and avoiding contextual factors and backdrops. Several significant and pivotal factors were down- or under-played in reporting on this tragedy. For example, the relationship to the Saudi Kingdom, human rights, billions upon billions of dollars in armaments sold to the Saudis, the unimaginable assault by Saudi Arabia against Yemen, and the impending famine and genocide in Yemen as a result, women’s rights, journalistic freedom, and an unending series of beheadings by the quasi-untouchable Saudi regime. Undoubtedly, information, discussion, debate, reports, and mobilization on all of these fronts can be located and advanced through social media, in spite of the mainstream, hegemonic vision. The point here is that central, controlled, and ‘manufactured’ debate, at least within a condensed and constrained optic and timeframe, shined a light on the actual killing of Khashoggi in Turkey, who did it, how, and why. Yet, significantly, it was only weakly concerned with the other, what could/should be considered to be, highly pertinent and central issues that are/were intertwined within this quagmire. Why such deference was paid to the Saudi leadership in this case, when this same deference is not paid in many other instances, especially when the faulty regime is not an ally, is quite pertinent.

The lack of historical, political, and economic context, combined with the propensity to avoid latching onto ‘research’, and a plurality of visions, perspectives, and experiences seem to be a predominant feature of how these stories crystallize. The Khashoggi example, like others, contains an evolving set of circumstances and frames, as well as questions, and we are cognizant of how some segments of social media can provide differing narratives that can, consequently, re-shape the ‘official’ story. Yet, the social media dimensions can also counter the formal hegemonic narrative, and this is where alternative forms of ‘democracy’ can start to take hold (Jenkins, Shresthova, Gamber-Thompson, Kligler-Velenchik, and Zimmerman 2016).

Why the more critical dimensions within the Khashoggi case (or the Venezuela situation or others, for that matter) were/are not more broadly taken up by Democracy 1.0 relates, I believe, to the hegemonic shaping/framing of the issues. It is also combined with a weakly focused mainstream media, whose reach is now consumed within the ‘fake news’ bubble, and a still questionable place, at least among many formal political leaders and their business sector supports, of uncontrolled social movements and social media within formal political spheres. However, I do believe that this last factor—social movements and social media being a mobilizing force—is, and will continue to potentially be, central to conceptualizing, developing, cultivating, building, and elaborating a more decent, meaningful, robust, and critically engaged democracy, in spite of the *status quo* aiming to maintain and sustain its hegemonic place.

Social media movements can also lead to dictatorship, genocide, and an infringement of rights (Sapra 2020). For instance, Gayo-Avello (2015) hypothesizes that social media may contribute but is not the central feature to democratization:

In short, social media is not a democratizing catalyzer per se. It is just one of many factors, in addition to great tactical tools, provided the conditions in the nondemocratic country are suitable. Moreover, there are many variables which can negatively affect the outcome of any uprising, even without the regime tampering with social media. In other words, social media does not make people free; freedom requires people taking risks and organizing themselves. (Gayo-Avello 2015: 6)

Social media cannot magically lead to class consciousness, anti-racism, peace, and social solidarity. However, it may be able to provide an outlet and legs to important stories, events, and realities for people who were only previously loosely connected. This could have a dual effect of further questioning and delegitimizing normative democracy, and also providing space and voice for marginalized interests, perspectives, and arguments. Social media is now indelibly a part of the citizen participation landscape.

### Neoliberalism and the Economics of Freedom

What is the point of living in a ‘democracy’ if you are one of those living in abject poverty, are homeless, and are working tirelessly to make ends meet but never achieve economic justice (Ely Yamin 2020)? Of course, the notion of having the ‘freedom’ to pursue your dreams, as in ‘the American dream’, is sufficiently grounded within normative debates to ensure that questioning entrenched, systemic, institutional, deeply grounded social inequalities will be quickly snuffed out. Within the USA context, Amadeo (2019) highlights the increasing social inequalities as follows:

Structural inequality seems to be worsening. Between 1979 and 2007, after-tax income increased 275% for the wealthiest 1% of households. It rose 65% for the top fifth. The bottom fifth only increased by 18%. That’s true even adding all income from Social Security, welfare, and other government payments.

During this time, the wealthiest 1% increased their share of total income by 10%. Everyone else saw their share shrink by 1–2%. As a result, economic mobility worsened.

The 2008 financial crisis saw the rich get richer. In 2012, the top 10% of earners took home 50% of all income. (Amadeo 2019)

Powers, Fischman, and Berliner (2016) have highlighted how research on poverty and social inequalities is poorly understood or operationalized, which further underpins weak policy responses to entrenched and systemic problems.

Similarly, it is helpful to problematize how wealth has been accrued historically through genocide, slavery, imperialism, war and conflict, colonialism, and a host of racialized, sexist, and other machinations in addition to Piketty’s (2017) well-documented treatise *Capital in the twenty-first century*. McLaren (see Pruyn and Malott 2016) has highlighted Marx’s theory on surplus value and the limited mobility between the social classes, and the crushing blow of capital against labor; ultimately, the value of what is produced encounters hyper-inflation in the hands of investors, owners, and speculators without real production, which may seem locked into the days of children being exploited in coal mines over a century ago but there are still many parallels today. Giroux (2016) has coined ‘casino capitalism’ to label the politico-economic system that enraptures the vast majority of formal, and to varying degrees, informal activity that underpin mass exploitation. He further elucidates the danger of continuing on the one-way neoliberal path before us:

Neoliberalism has put an enormous effort into creating a commanding cultural apparatus and public pedagogy in which individuals can only view themselves as consumers, embrace freedom as the right to participate in the market, and supplant issues of social responsibility for an unchecked embrace of individualism and the belief that all social relation be judged according to how they further one’s individual needs and self-interests. Matters of mutual caring, respect, and compassion for the other have given way to the limiting orbits of privatization and unrestrained self-interest, just as it is has become increasingly difficult to translate private troubles into larger social, economic, and political considerations. One consequence is that it has become more difficult for people to debate and question neoliberal hegemony and the widespread misery it produces for young people, the poor, middle class, workers, and other segments of society– now considered disposable under neoliberal regimes which are governed by a survival-of-the fittest ethos, largely imposed by the ruling economic and political elite. (Giroux 2016)

McLaren (see Pruyn and Malott 2016) and Giroux (see Giroux, Sandin, and Burdick, 2018) have also made a compelling case to interpret today’s reality as a politico-economic context that is launching us into hyper-sophisticated forms of fascism. Within this backdrop, I believe that there is a great need, as there always has been, to be more fully engaged with (and in) education, in political circles and in public debate, in general, in relation to the philosophy and operationalization of capitalism and, in particular, to the all-encompassing mercantilization of all public and private goods, services, and experiences enveloped within neoliberalism. The Covid-19 context has expedited and underscored the slippery slope toward authoritarianism, stripping away rights while creating socio-economic cleavages that are even more serious than before (Giroux 2020).

Democracy 2.0 is tethered to Democracy 1.0 conceptualizations of the world, but the door is (slightly) open to develop a new world, despite the titanic hegemonic vice-grip that maintains a stranglehold on education and public debate. As alluded to in the previous section, the collective ‘we’ are free to surf the web, consume, create, diffuse, comment, and cajole the other, whether the ‘other’ knows us, sees us, or cares about us or not. We are not frontally impeded from opening our eyes and ears. On the contrary, many movements have been stimulated from doing so—including the Arab Spring— although the aftermath re-captured regressive hegemonic features of what preceded it. The dilemma is that the corporate/business politico-economic (hegemonic) world has grown into this concurrently in-your-face and stealth, quickly-evolving, dynamic context seamlessly stamping its imprint in every way possible. The interplay between Democracy 1.0 and Democracy 2.0, thus, offers tremendous potential for citizen participation and engagement while, simultaneously, presenting the quicksand mirage that we may not be as ‘free’ as we think we are, or we may not be as ‘engaged’ as we think that we are. Neoliberalism has many people around the world gasping for air.

### Covid-19 and the Urgency to Avoid Returning to ‘Normal’

Now mired in a pandemic that vacillates from signs of encouragement that the ‘curve is flattening’ to fears that ‘community transmission’ is rapidly spreading through asymptomatic contact, there is enormous stress about when there will be an effective vaccination, how the health context will play out, and, increasingly, when will the ‘economy get back to normal.’ At this point in time—although we are aware of massive numbers (the information is not hidden, anyway) of unnecessary deaths in ‘developing’ countries related to hunger, disease, poverty, and conflict— we can see the extreme concern within local, national, and international governments and institutions to get the economy working. While most of the world has emphasized ‘social distancing’ as a key measure to diminish the dissemination and transmission of Covid-19, an eerily bizarre phenomenon has taken hold in the USA (Wong, Vaughan, Quilty-Harper, and Liverpool 2020). Disparate, semi-organized protests against ‘self-isolation’ are taking place in diverse locations, often replete with a range of arms and placards enunciating the right to, among other things, ‘haircuts’ and to ‘play golf.’ Is it pure insanity, a case of hubris beyond all limits, an anti-science ideology that needs to play out in every sector—including the environment—or complete indifference to human suffering? While the USA situation deeply underscores the anxiety and agitation around the health/economy dichotomy, I present below a brief illustration of the neoliberalization of the political and economic convergence through an example of the Coronavirus in Québec (Canada).

Québec, a predominantly French-speaking province of 8.5 million people in Canada, provides an interesting illustration of how a jurisdiction within a federal framework has worked to mobilize, sensitize, and activate a range of health, economic, political, and education measures to confront Covid-19. There are daily press briefings, information sessions, directives, a vast media campaign, testimonies, and a host of consultations, which all serve to educate the public and to engage the citizenry concurrently. It would be disingenuous to simply criticize where there have been gaps and problems; the reality is that many people have worked diligently and courageously to create a sense of the gravity of the problem and to diminish the extent of the propagation of the virus. Having a universal healthcare system has been, I believe, indispensable to understanding how to assess, allocate, distribute, and organize resources. This is not an individual problem but a vast, insidious collective one. It should be acknowledged as well that what we know is shifting and re-calibrating in real time, and decisions made on March 1 were questioned and re-assessed by March 7 and so on. Moreover, what we know now cannot always be fully understood until later, and decisions that are taken in that light can lead to nefarious situations and the rampant spread of the virus. Hindsight is 20/20 as the proverb goes so a fulsome diagnosis of what we are doing today will be more effectively critiqued once we are through to the other side of the pandemic.

The situation in Québec, one that is surely not unknown elsewhere, underscores the fragility of ‘normative’ democracy; this is, I believe, a question of normative democracy working the way that it does. One heart-wrenching issue that we are observing at this time is that the vast majority of deaths in Québec, like elsewhere, is among those 70 years of age and older, and particularly the 80 + age-group. Moreover, what many of us did not know or fully consider, the vast majority of deaths up until now within Québec are among those who are in long-term care residences (in French, they are called CSHLDs), roughly 70%, which are essentially senior’s residences for people with health issues. The transmission within these residences is extensive and rapid, with an increasing number of personnel, nurses, and doctors also being affected.

One residence, for example, experienced an overwhelming amount of infection (Herron, discussed below), and there are others that have also been deeply affected. One might say that there are two public health crises at this time: one for the general population and another for these particular residences with this specific group. On the one hand, the population is astonished, sickened, and in shock (‘How could this happen?,’ ‘Especially to “our elderly”?’). On the other hand, this was a serious politico-economic cocktail being mixed for a couple of decades, massaged through diverse political parties within the normative democracy that adjudicates such matters. (Why was there such sustained neglect and under-funding? Why was this not flagged as a serious catastrophe in the making?). I would like to underscore that this is not a problem of one person, one political party, one decision, one law, or one particular model: it is the consequence of systemic, institutional failure/negligence as well as the thinnest wedges of normative democracy carrying the day over the broader public interest and good.

I briefly present some of the specific underlying conditions that lay the groundwork for what is playing out within this vulnerable population at this time: a lack of monitoring, under-paying workers, and diminished policy importance and planning. Media accounts provide information on the tragedy unfolding before our eyes. In one case, at Herron, in Western Montréal, the CHSLD there, which is privately owned, experienced serious staffing shortages, insufficient equipment, poor oversight, inadequate support from oversight bodies, and unacceptable communications with health authorities. McKenna (2020) provides a sense of the chaos and suffering there:

Nurses were getting sick, too: six out of the seven registered nurses on staff were experiencing COVID symptoms, and of seven licensed practical nurses (LPNs), only four were still healthy. (…).

About a quarter of the orderlies (*préposés aux bénéficiares*, or patient attendants) had also stopped working — either because they were experiencing COVID symptoms or because they felt it was no longer safe to work at CHSLD Herron. Within weeks, a quarter of those patient attendants would test positive for COVID-19. (…).

Bedridden residents were lying in sheets stained brown up to their necks in excrement, so long had it been since their diapers had been changed. Some were dehydrated and unfed. (…).

The head of professional services at the CIUSSS, Dr. Nadine Larente, is the doctor who went to help. She told the French-language newspaper La Presse the place was in chaos: one LPN and two patient attendants were trying to care for 130 residents. Food trays had been placed on the floor, dishes untouched because residents with mobility issues could not reach them. (McKenna 2020).

About double the number, proportionately, of seniors in Québec opt for long-term care residences compared with the rest of the country, which could be a function of culture, policy, economics, and options available, and the rapidly aging Québécois population is a further aggravating factor preparing the context (Dougherty 2020). One expert (see Lowrie 2020) noted that the spatial configuration ‘with long corridors and residents sharing rooms, have a harder time isolating sick residents from uninfected ones, compared to residences with house-style layouts, where residents live in smaller wings’ is another factor that helps explain the extreme transmission of the virus in CHSLDs. With staff falling ill or refusing to come to work, there has been a massive campaign to recruit retired nurses and also to bring doctors and specialists into the overburdened long-term care system; the Premier of the Province has also asked for the military (over a thousand troops) to further provide support within these seniors’ residences.

Social class and political power are fully intertwined in the quickly unraveling situation involving seniors’ residences in Québec. Raising the minimum wage in Québec, for example, was vigorously opposed by the present government and others along the way, fearful that employers, especially small businesses, could not afford it. While there is no maximum wage being regulated, those struggling with the minimum wage are often obligated to work two or three jobs, to seek assistance elsewhere, and face other severe challenges, including in relation to housing, childcare, education, and the cost of living. The CHSLD situation brought everything to a head, with it being clearly obvious that those designated as ‘essential services’ were often those being paid the least in society. The Premier took the almost unprecedented measure of apologizing for underpaying workers when it became difficult and problematic to staff these residences:

‘I know a lot of Quebecers are asking themselves how we could have got ourselves in this situation,’ a sombre Legault said at his Friday briefing, addressing the catastrophe unfolding in COVID-19-stricken long-term seniors’ residences (CHSLDs).

‘I myself have spent several days and nights asking what I should have done differently.’

‘If I was able to redo one thing, I would have increased the wages of orderlies faster, even without the accord of the unions. I assume full responsibility. We entered this crisis ill equipped, and clearly the situation deteriorated for all kinds of reasons. The virus got in.’ (Authier 2020).

The Premier also took a series of steps to increase pay for healthcare workers.

As part of its effort to improve working conditions in the health-care system, Quebec announced that nearly 300,000 employees in both the public and private sector will be getting temporary pay increases.

Workers who are in direct contact with the disease — such emergency-room professionals and nurses in coronavirus testing centres — will receive an 8 % boost in their salaries.

Those working in long-term care homes, known as CHSLDs, will also be among the 69,000 workers to benefit from the 8 % raise….

Another 200,000 people who work in the health-care system but aren’t as directly exposed to the disease, such as the nurses who staff the 811 health line, will get a salary increase of 4 %.

And workers in private long-term care homes, many of whom make little more than minimum wage, will get an additional $4 per hour. That measure appears designed to discourage these workers from quitting and staying home, to take advantage of federal financial assistance that’s worth $2000 a month. (Shingler, Stevenson, and Montpetit 2020).

One question that arises here is how these workers could have been underpaid for so long, and what the effect may have been, for them, the people receiving the care, the healthcare institutions and system, and society as a whole. Did it dissuade qualified workers from pursuing careers or staying in them? What were the other priorities that negated remunerating fairly such indispensable and ‘essential’ workers?

On the economic side of the ledger, how efficient is it to underpay some employees and over-compensate others who have not actually done the work or who are, ironically, considered to be disproportionately fundamental? Is a 10:1 ratio for salaries at the top and the bottom reasonable or should it be 100:1 or 1000:1? In Canada, in general, the wage differentials are less extreme and odious than the USA, but the issue of social (in)equality is also a significant concern there. One study (Mishe and Wolfe 2019) focused on USA compensation provides some backdrop to how public services and priorities can be disproportionately affected.

CEO compensation is very high relative to typical worker compensation (by a ratio of 278-to-1 or 221-to-1). In contrast, the CEO-to-typical-worker compensation ratio (options realized) was 20-to-1 in 1965 and 58-to-1 in 1989. CEOs are even making a lot more—about five times as much—as other earners in the top 0.1%. From 1978 to 2018, CEO compensation grew by 1007.5% (940.3% under the options-realized measure), far outstripping S&P stock market growth (706.7%) and the wage growth of very high earners (339.2%). In contrast, wages for the typical worker grew by just 11.9%.

There is a lot of complexity to how Covid-19 is analyzed, and comparing diverse sites/jurisdictions/systems and how data are compiled and evaluated may not reveal the true breadth and scope of the reality. Similarly, there are many moving parts and lots of people (remunerated and volunteer) involved and engaged, and there are also all kinds of activities aimed at supporting a solidified, vigorous response. My intention in presenting this case study is not to admonish or diminish those serious and important efforts. On the contrary, it is my hope that this pandemic will reveal a silver lining somewhere in that extreme vulnerabilities and shortcomings need to be rectified in order to ensure, as much as possible, that economics will not suffocate political considerations in the future. And I have not emphasized here the race, gender, and other pivotal underlying factors underpinning this pandemic, but they are also a significant piece of the puzzle.

## Conclusion

This text has underscored what ‘democracy’ we are trying to achieve, to cultivate, and to ingratiate. The focus and direction of my central arguments about the lack of *bone fide* democracy within a normative, mainstream political framework that preaches that we live in a developed democracy has, I believe, become accelerated and accentuated as a result of Covid-19. I have highlighted some of the fundamental issues and problems with ‘normative, representative, hegemonic, electoral democracy,’ and also emphasized the pivotal contextual shifts and cornerstones embedded in Democracy 1.0 as well as Democracy 2.0. I have also made the case for more robust, critically-engaged citizen participation, which would require or, at the very least, benefit from new forms of education and media/political literacy. The social media equation was brought to light since it serves as an unruly, uncontrolled, and rapidly evolving microcosm of the world, its diversity, its problems, its challenges, and its potential. I was careful to not make a definitive declaration related to achieving democracy through the potentially transformative technologies that now shape how we live and function and relate to the world. Despite everything, we are still mired in conflict(s), in inequitable power relations, and in ‘democracies’ that are not very ‘democratic.’

We are still straddling Democracy 1.0, in which formal political declarations are fabricated with partisan political interests at the fore, the stock market is seemingly central to everything, and business elites are catered to at every level. Similarly, tax cuts—regardless of political stripe—figure into everything, political parties shamelessly line up to receive ‘donations’ (does anyone believe that they come with no strings attached?), tax breaks for companies must be considered as much as lower tax rates for the rich (does anyone believe that rich people will create more employment based on having more cash? If so, why are there so many off-shore accounts in tax-havens intended to not pay tax?), and (military) might is (still) right for many. The further the Coronavirus expands, the more there is discussion about needing an economic balance to ‘get back to normal,’ and indicators such as the stock market are central to supposedly gaging what is happening (Karabell 2020).

Of course, there have been lots of (incremental) changes, and lots of new laws, policies, practices, and shifts in cultural norms that have benefitted, generally speaking, women, racialized minorities, the poor, and the vulnerable. Yet, social inequalities, despite massive technological and others changes, not only persist but, in many regards, are increasing. How could this be when there is so much wealth? Why do so many people leave their countries in complete desperation, why is there still so much military conflict—most of which goes unreported—why do so many problems of poverty and discrimination persist in the most vulgarly palpable ways, why is there such little global outrage over the state and fate of Indigenous peoples (the loss of land, language, culture and autonomy), and why is the ‘environment’ not the priority? This very partial list of questions is noteworthy because neoliberalism is, definitely, an accelerator to many of the problems we are facing (Giroux 2020). To be clear here, this is not a binary proposition, and avoiding confronting real problems with real people will not address real suffering, oppression and marginalization (Gray and Gest 2018).

We might ask: Why are there (recurrent, entrenched) problems when there are so many people, projects, forces, and movements fighting for a more decent, robust, and (even) alternative Democracy 2.0, one that could place neoliberalism within a new, different and alternative landscape? How should hegemony be understood today when (many) people so freely believe that they have complete agency over their actions, thoughts and experiences, and when (many) people believe that voting is the (only) key? I would stress here that the binary capitalist-socialist, rich-poor binary is not the most productive lens through which to examine the complexity of such extreme power imbalances around the world. The debate around ‘democracy,’ I believe, needs to be more all-encompassing, involving all of the tentacles and blockages of neoliberalism into the class, race, gender, cultural, and other pivotal sociological markers of identity, and it also needs to carve out a place for how power works, is distributed and re-created. This debate needs to leave open the door for unknown questions and answers as well as (creative and alternative) processes and deliberations, accepting that the normative elections in place are most likely not very beneficial for most people, and, most definitely, the massive numbers of people who do not participate, willingly or unwillingly (Van Reybrouck 2017).

It is important to connect the local with the global, as we can through Covid-19. Ely Yamin (2020) provides a sense of the need to address global issues globally and to be leery of not considering the complexity of the linkages between complex problems.

But that and many other challenges requires weaving human rights praxis—human rights for social change—into broader social movements, as well as working across disciplinary silos. The problems facing the rights movement are too complex for any one set of advocacy tools or any one field’s expertise. Of course, there is no single monolithic ‘human rights community’ just as there is no unified ‘health and human rights community’. Those tropes are used from the ‘inside’ to police the boundaries of orthodoxy and from the ‘outside’ to caricaturize sets of actors and strategies. Yet, there are dangers of circling the wagons defensively around our professional tribes. The complexity of the challenges posed by rampant inequality, the spread of authoritarianism and illiberalism, distrust in multilateralism, and climate cataclysm call for embracing justified critiques and opening up to new ideas and perspectives—and uniting with labor, environmental and many other social justice movements. (Ely Yamin 2020)

Inspired by Paulo Freire’s transformative work (Freire 1970), I would advocate for more openness and acceptance of political realities that shape our lived experiences as well as an extremely healthy dose of humility as means to being able to understand, engage with, and be with the ‘other.’ I explore more fully the interconnections and inspiration of Freire’s work with my colleague Gina Thésée (Carr and Thésée 2019). The hard-wired, testosterone-induced, keep-fundraising-at-all-times political systems that have been put in place all over the place need to be re-imagined before they suffocate themselves and everyone else. People will slowly divest themselves from the voting game, leaving it as an empty shell filled with a bunch of White guys in suites. (Yes, there are some openings for other identities in this equation but the game was made by and for these guys.) Freire wrote of conscientization, and I believe that to get there, we need to focus on peace, not war, social, and cultural development as opposed to economic development, solidarity, and emancipation rather than exclusively on individual rights and liberties, and the recognition that we are (all) human beings. As human beings, we are not required to be racist (no baby is racist but we learn to be so), sexist (a totally learned behavior), classist (exploiting one’s neighbor is not an obligation), kill one another (who gets killed anyway? the rich or the poor, and who are they? do we care?), or live with so much misery, hatred, and oppression. Ultimately, we are in the same boat (or world) together.

One could see the glass half full with lots of progress all over the place, and, yet, the empty side of the glass contains real people living through unimaginable (for the full side of the glass) realities; the wage discrepancies and gaps in the Québec example exemplify this reality. The quest for a meaningful democracy aimed at both sides of the glass would be a more conducive option, and re-imagining democracy will require more fully and, even disproportionately, considering the empty side of the glass. Taking a stand against Democracy 1.0 and ‘normative, representative, hegemonic, electoral democracy’ is a necessary condition to moving forward for this re-imagined democracy.

Donkervoort (2020) underscores that the pandemic has been exploited by ‘autocrats’ but that citizens can resist and coalesce around global initiatives to weaken and confront hegemonic forces. This could mean enhanced civil society engagement across all boundaries with an eye to unmasking and dismantling the concentration of wealth and power. Covid-19 has exposed the need for a different universe, not only in terms of public health but also, importantly, in relation to democracy and citizen engagement (Roy 2020). So while my foot, to return to the title, may be in taters, I’m hopeful—indeed, it may be the only way out if this—that my head will not be the ultimate causality as we strive to either sustain or re-imagine a democracy that can not only take us out of a pandemic but, rather, into social solidarity that will remove our bodies and minds (and souls) from imminent disaster.
